# Isolation and Characterization of Canine Adipose-Derived Mesenchymal Stromal Cells: Considerations in Translation from Laboratory to Clinic

**DOI:** 10.3390/ani14202974

**Published:** 2024-10-15

**Authors:** Michael A. Rivera Orsini, Emine Berfu Ozmen, Alyssa Miles, Steven D. Newby, Nora Springer, Darryl Millis, Madhu Dhar

**Affiliations:** 1 Regenerative Medicine and Tissue Engineering, Department of Large Animal Clinical Sciences, College of Veterinary Medicine, University of Tennessee, Knoxville, TN 37996, USA; mriverao@utk.edu (M.A.R.O.); eozmen@vols.utk.edu (E.B.O.); snewby@vols.utk.edu (S.D.N.); 2 Genome Science and Technology, University of Tennessee Knoxville, Knoxville, TN 37996, USA; 3 Department of Biomedical and Diagnostic Sciences, College of Veterinary Medicine, University of Tennessee, Knoxville, TN 37996, USA; amilesc@utk.edu (A.M.); nspringer@utk.edu (N.S.); 4 Department of Small Animal Clinical Sciences, College of Veterinary Medicine, University of Tennessee, Knoxville, TN 37996, USA; dmillis@utk.edu

**Keywords:** canine, mesenchymal stem cells (MSCs), allogeneic, clinical applications

## Abstract

**Simple Summary:**

This study establishes in vitro criteria for the safe and effective use of allogeneic canine mesenchymal stromal cells for potential orthopedic and therapeutic use. Allogeneic canine adipose-derived MSCs demonstrated MSC-specific characteristics and met in vitro requirements, providing a solid foundation for future in vivo research and clinical applications. Importantly, their shipping and cryobanking conditions and related procedures were also tested and validated. This validation ensures the establishment of an allogeneic canine adipose-derived mesenchymal stromal cell bank that can be used as an off-the-shelf therapy.

**Abstract:**

In allogeneic MSC implantation, the cells are isolated from a donor different from the recipient. When tested, allogeneic MSCs have several advantages over autologous ones: faster cell growth, sufficient cell concentration, and readily available cells for clinics. To ensure the safe and efficient use of allogeneic MSCs in clinics, the MSCs need to be first tested in vitro. With this study, we paved the way by addressing the in vitro aspects of canine adipose-derived MSCs, considering the limited studies on the clinical use of canine cells. We isolated cAD-MSCs from canine falciform ligament fat and evaluated their viability and proliferation using an MTS assay. Then, we characterized the MSC-specific antigens using immunophenotyping and immunofluorescence and demonstrated their potential for in vitro differentiation. Moreover, we established shipping and cryobanking procedures to lead the study to become an off-the-shelf therapy. During expansion, the cells demonstrated a linear increase in cell numbers, confirming their proliferation quantitatively. The cells showed viability before and after cryopreservation, demonstrating that cell viability can be preserved. From a clinical perspective, the established shipping conditions demonstrated that the cells retain their viability for up to 48 h. This study lays the groundwork for the potential use of allogeneic cAD-MSCs in clinical applications.

## 1. Introduction

Mesenchymal stromal cells (MSCs) are multipotent stromal cells that can be derived from any adult somatic tissue, such as adipose tissue, amniotic fluid, amniotic membrane, bone marrow, umbilical cord blood, umbilical cord tissue, dental pulp, liver, muscle, olfactory epithelium, omentum, ovary, periodontal ligament, periosteum, placenta, synovium, peripheral blood, and Wharton’s jelly [1,2,3,4,5]. MSCs are typically derived from the mesodermal germ layer, with a high potential for self-renewal and in vitro trilineage differentiation [6,7,8]. Clinically, MSCs are being used widely as a therapeutic treatment for a multitude of injuries or diseases in veterinary medicine. MSCs have immunomodulatory functions [9,10,11,12,13], and both differentiation and paracrine signaling are implicated as mechanisms through which they improve tissue repair and differentiate into healthy cells in damaged or diseased tissue [14,15]. MSCs exhibit immunosuppressive and anti-inflammatory abilities which can accelerate tissue regeneration and attenuate inflammation. MSCs’ natural immunomodulatory function can, directly and indirectly, activate the apoptosis of T cells in the surrounding tissues to control inflammation [16,17]. Mediators and cytokines within an inflammatory environment activate MSC to release immunosuppressing mediators, such as IFNγ and TNFα, which results in the downregulation of inflammation by reducing lymphocyte production [9,10,13,18,19]. Additionally, in allogeneic transplantation, a lack of MHC-II expression alters antibody production, and co-stimulatory molecules escape the recognition of T Cells and NK receptors, thus regulating the immune response [20,21,22,23]. Adipose tissue and bone marrow are the two most favorable options for obtaining MSCs [24,25,26,27,28]. Adipose tissue can be relatively easily acquired during routine surgery, such as castration or ovariohysterectomy. In the case of ovariohysterectomy, adipose tissue can be collected from various locations, including the fat surrounding the falciform ligament and the broad ligament, as well as the peritoneal fat and mesenteric fat, which are all examples of white adipose tissue [29]. 

Clinically, MSCs can be used in an autologous or allogeneic manner [30,31]. In an autologous mode of implantation, the donor and the recipient of MSCs are the same animal, whereas in allogeneic implantation, they are different. Thus, autologous MSCs can be used without any characterization or testing. Allogeneic cells, on the other hand, require both in vitro and in vivo evaluations prior to use [32,33]. We have reported previously that horses treated with >90% CD90 positive and <2% MHC II negative expression equine allogeneic MSCs showed adverse reactions in only 4.35% of the cases [8,34,35]. As a result, we stated that equine allogeneic MSCs positive for CD90 and negative for MHC II can be used safely in the treatment of musculoskeletal injuries. Ongoing studies and a retrospective clinical trial in horses further support our claim [34]. A similar claim for canine MSCs is lacking. Hence, potential in vitro guidelines establishing criteria for the safe and efficacious use of canine MSCs should be established.

While the use of MSCs in both human and veterinary medicine has increased significantly over the years, challenges associated with the heterogeneity of MSCs need to be addressed before they can be routinely used to treat clinical cases [36]. The MSC heterogeneity is mainly associated with a lack of a uniform nomenclature to describe MSCs [23], differences in MSC behavior in vitro during expansion, and differences in MSC properties from different donors and tissues [4,7]. For instance, adipose-derived MSCs yield a higher quantity of cells and proliferation potential, while cell proliferation abates in sources such as bone marrow and amniotic tissue [4,37]. The effect of senescence on MSCs also varies depending on their source. For example, prenatal tissue is the least affected, while bone marrow is significantly affected [37,38]. Cellular size at early passages has been shown to be one of the factors that impact cellular proliferation and senescence [39]. Moreover, tri-lineage differentiation differs between sources; for instance, adipose-derived MSCs present an increase in genetic expression to adipogenesis, while bone marrow-derived MSCs have an increased expression toward osteogenesis [40]. Although there is higher expression between sources, adipose-derived and bone marrow-derived MSCs can undergo tri-lineage differentiation. Tri-lineage differentiation potential in vitro is influenced by cell culture media supplements and growth conditions [40,41,42]. 

These in vitro variations can potentially lead to variable outcomes in clinical cases for various conditions, leading to questions by practitioners regarding the proper use of MSC-based therapies. One way this issue can be resolved is by performing a thorough evaluation of MSC properties in vitro and then translating them in vivo. This approach is possible if we use the allogeneic mode of implantation as an off-the-shelf therapy. Under proper cryopreservation storage conditions (−80 °C and colder), cells maintain their viability for over 10 years [43,44,45]. For an off-the-shelf therapy, allogeneic MSCs are readily available at a moment’s notice to be used, can be grown from different sources, and have low antigenicity [46,47]. The process for off-the-shelf therapy requires cells that can proliferate, undergo tri-lineage differentiation, are immunomodulatory, and show expression of MSC markers while lacking antigen-presenting proteins, such as MHCII [31,34,48,49]. After meeting these criteria, these MSCs must be tested for cryopreservation to allow long-term storage while maintaining viability and function after being stored. Cellular viability after cryopreservation allows the MSCs to remain stable under proper shipping conditions.

It is possible that allogeneic MSCs may cause immune rejection and immunological memory, as well as transmit disease-candidate genes [50,51]. Disease-candidate genes are a disadvantage for both allogeneic and autologous MSCs [52]. Autologous MSCs do not carry the risk of immune rejection when used appropriately, but they result in a longer waiting time for cells to grow and obtain a sufficient concentration for implantation. Additionally, the patient’s health status can result in poor MSC proliferation and differentiation [53]. This results in a lack of assurance that these MSCs will grow from any patient’s sample. 

Currently, autologous stromal cells are used primarily in canine MSC therapy. However, autologous MSCs may carry donor morbidities affecting cell proliferation. Also, current therapies lack detailed characterization of cell surface markers, specific quality control parameters, and manufacturing processes [54]. Given all of these challenges and limitations in current canine MSC therapies described above, and in view of the need for canine allogenic MSCs, which can be readily available to the clinic, we conducted the present in vitro study. 

In this study, we aimed to isolate and characterize allogeneic canine adipose-derived mesenchymal stromal cells (cAD-MSCs) and establish proper expansion, handling and storage conditions for the cAD-MSCs to create an off-the-shelf therapy for canine injuries Our long-term goal is to use the characterized cells safely and consistently in the clinic. We hypothesize that well-characterized allogeneic MSCs that are viable under specific storage conditions, and that exhibit positive and negative expressions of specific protein targets, are strong candidates for further in vivo and clinical trials. To establish the groundwork for testing the hypothesis, we isolated MSCs from adipose tissue, carried out proliferation, immunophenotyping, and differentiation assays, and finally generated a cryobank of well-characterized MSCs for clinical applications.

## 2. Materials and Methods

### 2.1. Methods

All biochemical, chemicals, and disposable materials were purchased from Thermo Fisher Scientific (Waltham, MA, USA) unless noted otherwise.

### 2.2. Isolation and Expansion of Canine Adipose-Derived Mesenchymal Stromal Cells (cAD-MSCs)

Mesenchymal stromal cells were isolated from falciform and broad ligament fat collected from a 21.7 kg healthy 13-month-old female Pitbull mix during a routine ovariohysterectomy procedure. The owner’s consent was obtained prior to collection of the tissue. This protocol was exempt from the Institutional Animal Care and Use Committee (IACUC) because the tissue is deemed biomedical waste that was opportunistically collected during a routine procedure.

Approximately 35 g of adipose tissue was collected. The tissue was dipped into 1% bleach for 2 s to reduce contaminants and was then generously washed in sterile phosphate buffer saline (PBS) to neutralize the bleach. Subsequently, the tissue was transported (within 30–40 min) in a cooler to the laboratory for processing.

The adipose tissue was minced thoroughly using a sterile #10 scalpel blade in the presence of Hank’s Balanced Salt Solution (HBSS) at room temperature and then was enzymatically digested using collagenase Type I (2 mg/mL) in HBSS. 1 g of tissue was digested with 1 mL of collagenase solution in an orbital 37 °C shaker for one hour. In order to maximize enzymatic digestion, the conical tube was placed at an angle of approximately 45° with the shaker set at 300 rpm. The mixture was checked every 15 min until the tissue was digested and no visible tissue pieces were seen. The mixture was slowly passed through a 100 µm filter into a 50 mL conical tube to remove tissue debris. The filtrate was further centrifuged at 190× *g* for 10 min at 15 °C. The top two-thirds of the supernatant was discarded. The remaining sample was seeded in four T-75 flasks in growth media consisting of Dulbecco’s Modified Eagle Ham’s F12 medium (DMEM-F12, Cytiva, Marlborough, MA, USA) containing 20% fetal bovine serum (FBS), 1% penicillin, and streptomycin (10,000 U/mL), and 1% amphotericin B (250 µg/mL). Flasks were incubated at 37 °C in a humidified incubator containing 5% CO_2_ for seven days before changing media to 10% FBS DMEM-F12. Colonies of cAD-MSCs appeared within this time. The media containing 10% FBS was used as the growth media thereafter.

The cAD-MSCs were expanded in the growth media by incubation at 37 °C and 5% CO_2_ with media changes every 2–3 days until the cells reached 80–90% confluency. Confluent cells were washed twice with HBSS and collected by treating cells with 0.25% trypsin-EDTA for 5 min at 37 °C. After 5 min, the enzymatic activity was stopped by adding an equal amount of growth media. Cells were collected by centrifugation at 300× *g* for 10 min. Cells were counted using the Countess 3.0 (Invitrogen, Carlsbad, CA, USA) and were either cryopreserved or used for further experiments. 

Cells were expanded through passage six. Aliquots of cells were cryopreserved at every passage. For cryopreservation, one million cells were resuspended in 1.5 mL of cryoprotectant solution, composed of 50% FBS, 45% DMEM-F12, and 5% dimethyl sulfoxide (DMSO) (Sigma Life Science, Burlington, MA, USA). Cells were placed at −80 °C overnight inside a controlled cryopreservation chamber and then transferred into liquid nitrogen for long-term storage.

### 2.3. MTS Assay

Cellular proliferation of the cAD-MSCs was quantitatively evaluated using the CellTiter 96^®^ AQueous One Solution Cell Proliferation Assay (Promega, Madison, WI, USA), as described earlier [34,35]. Approximately 20,000 cells of passage 2 were seeded per well in a 24-well plate. Samples were set up in triplicate. An MTS assay was carried out on days 2, 4, 6, 8, and 10. For the assay, the MTS reagent (5:1 media: MTS reagent) was added, and samples were incubated at 37 °C for 3 h. Total absorbance was measured every two days using the Synergy^TM^ Ht Microplate Reader (Biotek, Winooski, VT, USA) at an absorbance of 490 nm. Data was normalized using cell growth media as a control, and the graph was plotted to represent the absorbance versus time.

### 2.4. Immunophenotyping of cAD-MSCs by Flow Cytometry

The quantitative expressions of antigens known to characterize MSCs were analyzed by flow cytometry as per the methods reported earlier [35]. Passage 2 cells were collected and were evaluated for the expression of CD44, CD90, CD45, MHC I, and MHC II antibodies previously validated in the dog and listed in Table 1. Approximately 1 × 10^6^ cells were used per staining reaction per marker in single-color tubes. 

A combination of direct (CD44, CD45, MHCII) and indirect methods (CD90, MHCI) of staining was used. An amount of 0.5–5 µg/mL of each antibody was used per staining reaction as recommended by the manufacturer. Primary antibody reaction was carried out at 4 °C for 60 min in the dark. For CD90 and MHCI, 2 µg of corresponding secondary antibodies were used. Samples were incubated at 4 °C for 15 min in the dark. Following staining, each sample was washed with PBS (1×), and cells were fixed using 4% paraformaldehyde (PFA) by incubation at room temperature for 15 min. Samples were stored at 4 °C until the analyses. Prior to analyses, 1 mL of Attune focusing fluid (1×) was added to each sample, and samples were analyzed on the Attune NxT Flow Cytometer (Thermofisher, Waltham, MA, USA). The cAD-MSC cell population was identified by FSC vs. SSC characteristics and gated to exclude cellular debris. Percent positive cells within the cAD-MSCs gate for each of the CD or MHC molecules was determined by overlaying unstained cells to account for background fluorescence.

The expression of a subset of specific markers listed in Table 1 (CD90 and CD44) was also confirmed by collecting P2 cAD-MSCs by scraping. Cells were collected non-enzymatically by scraping to investigate whether the lack of expression by immunophenotyping was due to the accessibility of the epitopes and not due to any other artifact. Flow cytometry immunophenotyping was performed as described above.

Canine peripheral blood-derived mononuclear cells were used to validate the expression of specific markers, including CD44, CD45, CD90, MHC I, and MHC II, using the methods described above. One mL of peripheral blood was collected from a healthy research dog in an EDTA-coated tube. Blood was submitted to the University of Tennessee College of Veterinary Medicine Clinical Pathology laboratory for routine analyses. Immunophenotyping was performed to ensure that the positive and negative expressions of specific proteins were an accurate representation of the expression in MSCs. Red blood cells were lysed by adding 3 mL of 1× eBioscience RBC lysis buffer (Invitrogen, Carlsbad, CA, USA) at room temperature for 5 min. The sample was centrifuged at 1000× *g* for 3 min, and the pellet was collected. Red cell lysis was performed twice. The pellet was resuspended in 1× PBS for immunophenotyping. Cells were stained as described above. For data analysis, samples were gated on lymphocytes. Expression values were analyzed using FCS Express 7. 

### 2.5. Immunophenotyping of Cryopreserved Cells by Flow Cytometry

Once the antibodies were validated for freshly expanded cells, the expression of specific markers was also evaluated in cryopreserved MSCs using the methods described above [35]. Briefly, cryopreserved cells were thawed as per the methods reported earlier and stained with specific antibodies as described above [35]. The cAD-MSCs were stained for markers listed in Table 1. This was performed to ensure that the expression of markers was not affected in the cryopreservation step.

### 2.6. In Vitro Differentiation

The in vitro differentiation potential of the MSCs was evaluated by differentiating passage 3 cAD-MSCs into adipocytes and osteocytes, as described earlier [35]. Expanded MSCs were seeded into a 6-well tissue culture, polystyrene-treated plate at a density of 2 × 10^5^ cells/well. Adipogenic differentiation was induced using complete growth media supplemented with 2.5 mM dexamethasone (DEX), 0.02 mM indomethacin (IND), 10 μg/mL insulin, and 0.5 mM 3-isobutyl-1-methylxanthine (IBMX). Media change was performed every 48 h until the cells were fixed at specific time points using 4% paraformaldehyde. Adipogenic differentiation was confirmed by staining with 0.7% *w*/*v* in 85% propylene glycol solution of Oil Red O as reported earlier [34,35]. Stained cells were imaged using a Leica DMi1 inverted microscope (Leica Microsystems, Wetzlar, Germany).

Osteogenic differentiation was induced in complete growth media supplemented with 1 μM DEX, 0.25 mM ascorbic acid (ASC), and 10 mM B-glycerophosphate (BGP). Osteogenic differentiation was confirmed by staining with alizarin red, as described earlier [35]. Stained cells were imaged using a Leica DMi1 inverted microscope (Leica Microsystems, Wetzlar, Germany).

### 2.7. Identification of the Vehicle for Suspending MSCs during Shipping

Cell viability was evaluated after suspending MSCs in saline and subjecting the suspended cells to shipping conditions. This experiment was done to establish a suitable vehicle for the cells for implantation, as well as to evaluate their tolerance during shipping. Roughly 10 × 10^6^ cells were removed from liquid nitrogen and were thawed rapidly (<1 min) in a 37 °C water bath, using the methods described previously [34]. All reagents were prewarmed to 37 °C prior to use. 20 mL of phenol red-free DMEM-F12 without any additives was added dropwise to the thawed cells. After mixing, cells were collected by centrifugation and the pellet was suspended in 1.5 mL of 0.9% sodium chloride. Resuspended cells were transferred into a sterile 3 mL Luer lock syringe using a 20 g, 1.5″ hypodermic needle. A Luer lock cap replaced the needle to maintain sterility, and a 4″ × 4″ sterile gauze was used to cushion the syringe into a 50 mL syringe case. The syringe case was sealed using a 1″ strip of parafilm. It was placed into a 20.32 × 15.24 × 17.78 cm foam-insulated Styrofoam box with a 1.5″ wall thickness containing two 16oz ice packs. The box was then placed inside the trunk of a vehicle, which was left in a parking lot with temperatures reaching over 31 °C. Ambient temperature readings were taken using an Olympia infrared thermometer (#1485544) every 2 h for 6 h, followed by readings at 25, 48, and 75 h. At each time point, the syringe was inverted, and a 10 µL sample was obtained under sterile conditions. Cellular counts and viability were assessed using 0.4% Trypan blue solution with Countess 3.0 (Invitrogen, Carlsbad, CA, USA) at each time point. We tested shipping and cryobanking procedures to ensure that the MSCs did not undergo any change during these processes. We describe basic cell culture assays that are necessary to establish a very well-characterized and validated allogeneic stromal cell bank that can be used as an off-the-shelf therapy.

### 2.8. Immunofluorescence (IF)

#### 2.8.1. Detection of MSC Surface Markers

The qualitative expression of cAD-MSC surface markers, which were negative by flow cytometry, was also analyzed using immunofluorescence (IF) using the previously reported methods [57,58]. Briefly, approximately 2 × 10^5^ cAD-MSCs were fixed in a 6-well plate with 4% PFA at 24 h post-seeding. Then, 0.1% Triton X-100 was added for 10 min at room temperature to permeabilize the cell membrane. Finally, samples were rinsed twice with HBSS and were treated with a universal protein block to reduce the nonspecific binding of antibodies. Samples were incubated for 30 min at room temperature. Following the universal protein block, samples were incubated at 4 °C overnight with 4 µg CD90, 5 µg CD44, and 2 µg CD29, using protein block as a diluent. Subsequently, samples were washed with HBSS (2×) and incubated for 20 min at room temperature with their respective secondary antibodies. One drop of Prolong Gold antifade reagent with 4′, 6-diamidino-2-phenylindole (DAPI) was applied to each well to visualize cell nuclei, and a coverslip was placed over the cells. Imaging was done the next day with a Leica DMi8 fluorescence microscope at (10×) magnification (Leica Microsystems, Wetzlar, Germany).

As listed in Table 2, the primary antibodies Collagen I, Collagen II, Fibronectin, Vimentin, and Vinculin used in IF are generated against human proteins.

#### 2.8.2. Cytoskeletal Organization and Expression of Extracellular Matrix (ECM) Markers

Immunofluorescence was also performed to evaluate the cytoskeletal organization of the cells and the expressions of ECM markers using the methods described above. The expression of collagen I, collagen II, fibronectin, vimentin, and vinculin were evaluated. Alexa Fluor conjugated phalloidin was used to stain F-actin and was used to evaluate the cytoskeletal organization of cells adhered to the tissue polystyrene surface. All procedures were as reported earlier by Newby et al. [58]. The antibodies used to show the cytoskeletal organization and expression of ECM markers are listed in Table 2. All primary antibodies were validated for their cross-reactivity to cAD-MSCs using secondary antibody controls.

## 3. Results

### 3.1. cAD-MSCs Were Obtained, Expanded In Vitro, and Cryopreserved

Allogeneic canine adipose tissue-derived MSCs were generated for potential use in the clinic. We first evaluated the expansion and cryopreservation of cells in culture. Roughly 30 g of collected tissue resulted in 1.5 × 10^5^–2 × 10^5^ cells/cm^2^ after 4 days. These were further expanded by seeding roughly 1000 cells/cm^2^. After 7–10 days of expansion in culture, 8 × 10^4^ cells/cm^2^ were obtained, confirming that the cells were proliferating and would provide enough to cryobank. After the initial passaging, cells were routinely expanded by seeding 2 × 10^5^ cells in a T175 flask. Culturing them for 6–7 days typically yielded 8–10 × 10^6^ cells per flask for cryobanking. This process was repeated until passage 6. Results also provided us with guidelines on cell expansion, i.e., cell numbers to seed in a given area and the timing of their confluency. This data will help clinicians schedule cases for treatment with allogeneic MSCs.

At each passage, cells were evaluated to ensure that they adhered to the polystyrene surface of the flasks with spindle-shaped morphologies. We observed that around passage 6, cell morphologies started to change, suggesting changes in the cells; as a result, cells were cryobanked only up to passage 6 [60], thus generating a primary cell line and a cryobank of canine cells (Passage 1–5) isolated from the stromal vascular fraction. The viability of the cryopreserved cells was also analyzed at each passage by reseeding cryobanked cells into a new tissue culture flask and expanding them in vitro. 

### 3.2. Passage 2 cAD-MSCs Demonstrated a Linear Increase in Cell Numbers with Time

**Figure 1:**
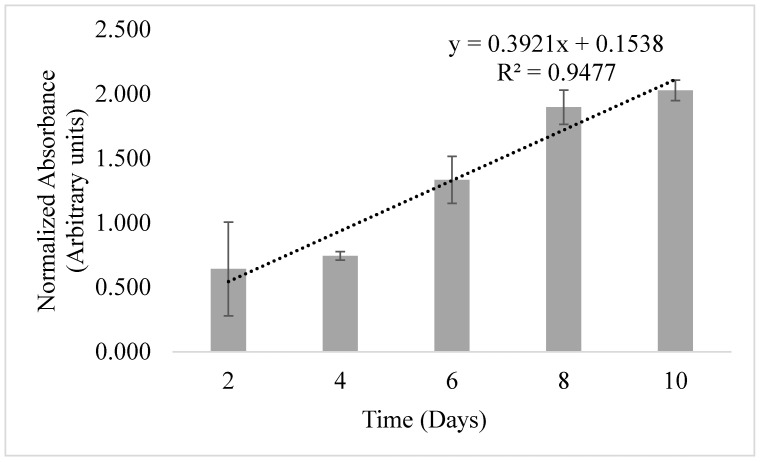
The proliferation of cAD-MSCs at passage 2 was assessed by MTS proliferation assay. Data were normalized using cell growth media alone as the control. Note the linear trend (R^2^ = 0.9477) in proliferation with time.

After we confirmed the expression of specific protein markers to prove that the cells are indeed MSCs, cell proliferation and expansion of passage 2 cells were quantitatively confirmed by MTS assay. The assay was performed on cAD-MSCs over 10 days. As demonstrated in Figure 1, passage 2 cells grew linearly with time, confirming their adherence to the tissue culture substrate and expandability and providing evidence of their viability during expansion.

### 3.3. cAD-MSCs Expressed MSC-Specific Cluster-of-Differentiation Markers

**Figure 2:**
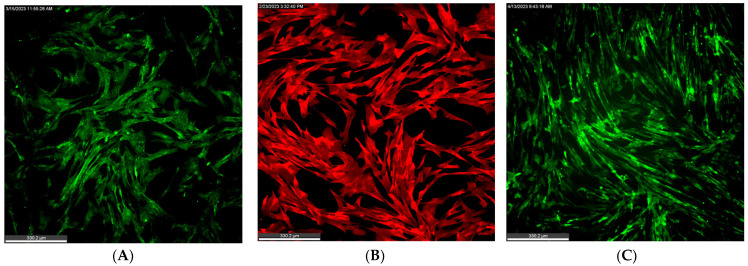
MSC surface marker expressions. Representative images of immunofluorescence show the expression of CD29 (**A**), CD44 (**B**), and CD90 (**C**). Cells were fixed and stained at 24 h post-seeding.

Using a combination of flow cytometry (using cells harvested by enzymatic and non-enzymatic treatments) and immunofluorescence, we confirmed that the cells isolated from the stromal vascular fraction and cryobanked were indeed MSCs (Table 3). As a result, we were able to generate a primary cell line of passages 1–5 of cAD-MSCs, which were positive for CD29, CD44, CD90, and negative for MHCII. It was intriguing to see the lack of CD29 and CD90 expression in enzymatically collected cells. Even though the expression of CD90 or the Thy-1 protein was originally associated with tumorigenic cell lines, it is considered as a minimal criterion for cells to be classified as MSCs [49,61]. CD29, or integrin β1, has consistently been demonstrated to be expressed in MSCs isolated from various sources and species [62]. Interestingly, we have previously demonstrated that an anti-rat CD90 antibody, Clone OX-7 (Invitrogen, Carlsbad, CA, USA), cross-reacted with equine MSCs and, as a result, served as a reliable marker in equine allogenic MSCs used as an off-the-shelf therapy for the treatment of soft tissue injuries in horses [8,34]. Hence, it was surprising to see the lack of CD90 expression in the cAD-MSCs on initial flow cytometry analysis. Interestingly, we observed 55.3% of cells to be CD90 positive when the cells were collected by non-enzymatic methods of scraping and IF, suggesting that cell handling can influence either expression or antibody labeling of this marker (Table 3). Additionally, IF showed a strong expression of both CD29 and CD90 (Figure 2), confirming that the loss of CD29 and CD90 epitopes could be due to the enzymatic treatment. Cell morphology and numbers, along with the expression of MSC markers, were also checked to ensure that cells did not undergo any changes during cryopreservation. Cells retained the expression of MSC markers (Table 3, Figure 2). Ultimately, using multiple techniques, we were able to show that cells were positive for CD29, CD44, and CD90, confirming that the cells are indeed MSCs (Table 3).

### 3.4. Cell Surface Marker Expressions Were Conserved between Cryopreserved and Freshly Expanded Cells

Freshly collected cells from the culture, as well as cells recovered from cryopreservation (at passages 1–5), were positive for CD29, CD44, and CD90. In contrast, they were negative for MHCII, confirming that MSCs did not lose their “stemness” markers during cryopreservation. Data also confirmed that our methods of harvesting cells from growing cultures and recovering them from cryopreservation did not affect the properties of MSCs.

Since the expression of MHC II is critical to deciding whether the MSCs can be used allogenically, we confirmed that the lack of MHCII expression was indeed a cellular property of cAD-MSCs and not due to the lack of antibody reaction. Cells derived from canine peripheral blood were used as a positive control [63]. As expected, cells showed the expression of CD44 (97.22%), CD45 (99.31%), CD90 (77.61%), MHC I (98.66%), and MHC II (20.642%), confirming the reactivity of each antibody. Specifically, roughly 20% of peripheral blood-derived cells were positive for MHC II, verifying that the lack of expression in cAD-MSCs was not an artifact. 

### 3.5. Adipogenic and Osteogenic Differentiation Patterns Were Observed at Passage 3 over 29 Days

**Figure 3:**
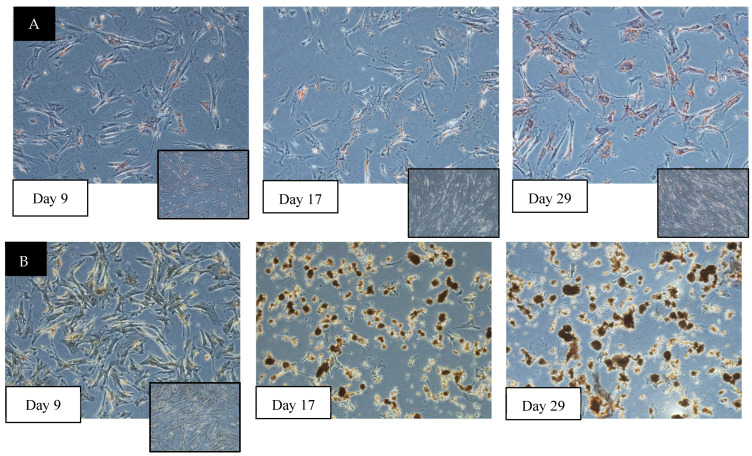
In vitro differentiation of cAD-MSCs. Representative images demonstrating the in vitro potential of cAD-MSCs to undergo differentiation. (**A**) Adipogenic differentiation was examined using Oil Red O staining. Note that the cell morphology and the Oil Red O-stained cells appear at around day 9 and continue to progress with time. (**B**) Osteogenic differentiation was examined using alizarin red staining. Note that the cell morphology and the alizarin red-stained cells appear as nodules around day 17 and progress with time. Nodules that are rich in calcium are the hallmark features of osteogenic differentiation. Insets show the corresponding undifferentiated control cAD-MSCs. These cells were maintained in normal growth media without any inducers.

In vitro differentiation of MSCs into adipocytes and osteocytes was performed over 29 days to evaluate the multipotent nature of cAD-MSCs (Figure 3). Subjective changes in cell morphology, along with the staining patterns of cells exposed to the differentiation media, were compared to the undifferentiated controls. Changes in the cellular structures undergoing adipogenic differentiation were first observed at day 9 post-differentiation. Comparatively, osteogenic changes were first observed after day 14. Based on special stains, differentiation was deemed complete by day 29.

### 3.6. Cells Retained Their Viability in Saline and during Shipping Conditions

**Figure 4:**
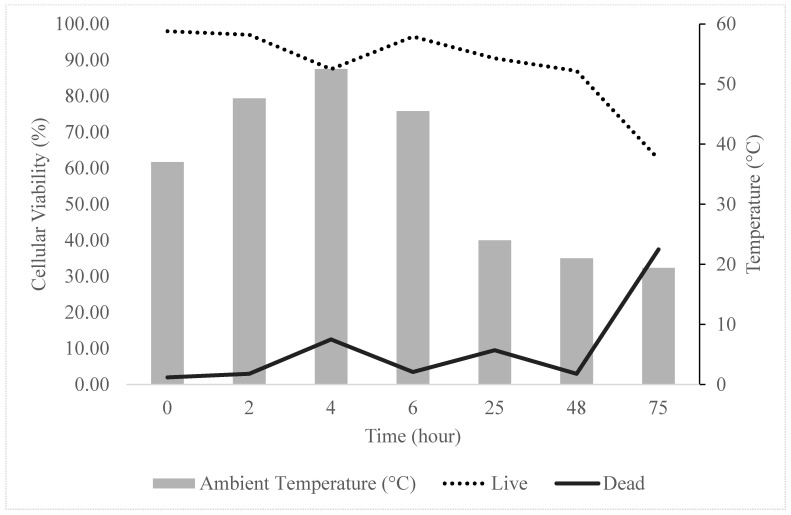
Percentage of live to dead cells over a period of 75 h in shipping conditions (ambient temperature of transport vehicle). The viability of cAD-MSCs was maintained in shipping conditions for over 48 h. Viability decreases at 75 h post-preparation.

As represented in Figure 4 and Table 4, we next evaluated the cell viability during shipping conditions. Cell viability of 10 × 10^6^/1.5 mL MSCs was maintained at >80% over a period of 48 h in saline. Thereafter, the viability was significantly reduced. Our data shows that cAD-MSCs can be safely shipped within the country and used within 48 h when packaged on ice and in the presence of saline as the vehicle. Saline is a commonly used and available solution in a clinic. Thus, we do not require any particular buffer or a solution to suspend MSCs for implantation.

### 3.7. Canine MSCs Adhere to Polystyrene Surface and Produce Extracellular Matrix

**Figure 5:**
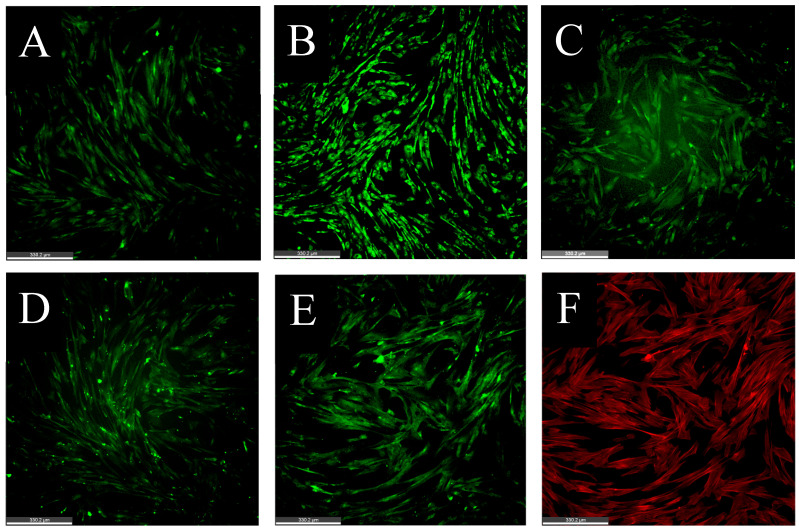
Extracellular matrix (ECM) protein expressions by immunofluorescence. (**A**) Collagen Type I. (**B**) Collagen Type II. (**C**) Fibronectin. (**D**) Vimentin. (**E**) Vinculin. (**F**) F-actin. Even though the ECM expressions are not the same between all these images from different proteins, they demonstrate the expression of all the ECM proteins.

Finally, thinking proactively about using the allogenic cells in tissue engineering strategies, i.e., combining MSCs with a suitable biomimetic scaffold for the treatment of any musculoskeletal injury or disease, we evaluated whether the MSCs had the potential to express specific ECM proteins when they adhere and proliferate on a suitable substrate. As shown in Figure 5, cAD-MSCs express collagen type I, collagen type II, fibronectin, vimentin, and vinculin when they adhere and proliferate on a tissue culture substrate. The expression of these proteins was observed within 24 h of cell seeding. Cytoskeletal organization was also confirmed via the expression of Alexafluor-tagged phalloidin staining of F-actin. The specific pattern in actin expression further confirms the cellular viability, morphology, and function.

## 4. Discussion

Adipose tissue and bone marrow are the two commonly used sources of MSCs in cell-based therapies. Although both sources of MSCs are similar, ultimately, it is the clinician or researchers’ choice to decide the method with which they are most comfortable. Adipose-derived MSCs are relatively easy to harvest and contain an abundance of MSCs [64,65]. Depending on the body condition score of the dog, harvesting both the bone marrow and the adipose tissue may be challenging. Comparatively, bone marrow may require a relatively invasive procedure of aspiration, which can result in complications when thrombocytopenia and neutropenia are present [66]. Mesenchymal stromal cells can be used in the clinic to treat injuries or diseases in an autogenic or allogenic manner. Autologous cells pose significant challenges in all species. For example, the quality and quantity of MSCs may vary with the donor’s age, source, and health [14]. As a result, autologous cells may be compromised in their rates of proliferation and function and, thus, may be ineffective in the clinic [14,67,68]. To improve clinical outcomes, it would be preferable to characterize MSCs prior to their use in the clinic. It is preferred to use MSCs as soon as a diagnosis is made in the clinic. As a result, having a cryobank of well-characterized MSCs, which can be made available to the clinic as needed, might enhance the use of cell-based therapies. 

Cell-based therapies, whether autologous or allogeneic, suffer from four major challenges—reproducibility, contamination, viability, and change in cell identity and, thus, function during the in vitro expansion process [69,70,71]. These parameters are affected by the number of cells harvested from the source tissue and seeded in culture for expansion. They are also affected by abnormal growth patterns due to inconsistent cell attachment and proliferation; frozen stock conditions; cell passage number; and time of growth to avoid under- or over-confluency, or merely by using the incorrect media and fetal bovine serum for cell growth. Uniform and in vitro validated cell culture conditions and parameters are essential for the success and reproducibility of cell-based therapies. A cryobank of well-characterized MSCs is thus a prerequisite for the success of stromal cell-based therapy in the clinic.

To our knowledge there are only two recent articles (Jong-Hyun et al. and Armitage et al. [72,73]) which are clinically relevant, and which describe the use of MSCs in clinical cases. This suggests that there are very few reports describing the in vivo clinical use of canine MSCs, possibly due to the challenges of cell-based therapies mentioned above. Allogeneic bone marrow-derived canine MSCs were used in two healthy dogs by Jong-Hyun et al. [72] for renal repair. Their study showed favorable glomerular filtration results after administering 3 × 10^6^ MSCs intraarterially directly into the right kidney. It was shown that these superparamagnetic iron oxide tagged-MSCs caused “inflammation, tubular necrosis, mineralization, and fibrosis without any functional complications” possibly due to the lack of testing for CD90 and MHC I or MHC II expression [74]. The authors did not report the expression of specific MSC marker proteins in the cells [75]. They used the cells in an allogenic manner but did not report the negative expression of MHCII either. This study could be stronger if the MSCs were characterized prior to their use. 

Mesenchymal stem cells have been tested for the expression of specific cell surface markers such as CD90, CD44, MHC-I, and MHC-II. It is imperative to demonstrate the expression of these targets when allogenic MSCs are used [34,50,76]. Moderate levels of MHC I and low levels of MHC II expression reduce adverse effects due to the lack of mismatch in MHC proteins [10,34,77]. It has also been shown in equine medicine that even when using autologous stem cells in limbs with reduced blood flow, such as in the joints, there were post-injection inflammatory changes or “flares” [78].

Armitage et al. cultured adipose-derived MSCs up to passage 4 and characterized them via flow cytometry. They used autologous MSCs that expressed CD44 and CD90 and did not express MHC II for treating chronic degenerative musculoskeletal conditions in 245 dogs [73]. This is acceptable. MSCs were characterized through differentiation, and the post-cryopreservation viability was ensured. The study demonstrated statistically significant improvement in range of motion after injecting 2.5 × 10^6^ cells into the affected joint intra-articularly. Adverse events, such as joint flare, were reported in less than 1% of the study population, lasting for less than 36 h. This study reported positive clinical outcomes using autologous MSCs, which is very encouraging. Our current study results in a bank of allogeneic MSCs ready to use off-the-shelf as needed for clinical therapy rather than prompting the patient to undergo additional procedures and waiting for sufficient cell quantities for treatment.

Regardless of the type of tissue collected to isolate MSCs, it is crucial to address and report their fundamental cell properties, characterizations, and storage methods to enhance clinical outcomes. In the current study, we report the basic cell properties of cryobanked cAD-MSCs, which can be used as guidelines in the choice of allogeneic MSCs as an off-the-shelf therapy for the treatment of clinical cases. Characterization of MSCs must occur before implanting them into an animal to minimize the risk that the MSC will not cause any adverse effects. Whether cells are used allogenically or autologously, they require these analyses to ensure that, when used, the MSC’s immunomodulatory function does, in fact, help the patient. Given the immunomodulatory function of MSCs [11], the cAD-MSCs under study here are not only potential candidates for future in vivo studies and clinical trials for canine injuries, but also hold promise as a candidate for anti-inflammatory therapies. In cell-based therapies, it is critical that the cells are characterized at different stages of proliferation; thus, using cell proliferation and morphological evaluation, we have characterized cAD-MSCs in multiple passages. We observed morphological changes beyond passage 6, indicating a potential change in their function. Using flow cytometry and immunofluorescence, we determined that the cAD-MSCs in our study did not lose the expression of specific surface markers or dissipate their proliferation potential, suggesting the potential for allogeneic use. A larger pool of donor dogs needs to be tested to ensure these findings can be extrapolated to cAD-MSCs more generally. The cAD-MSCs reported in this study had some inconsistencies in the expression of CD90. For this reason, we performed immunofluorescence on the cAD-MSCs, which showed expression of CD90. We replicated the immunophenotyping by flow cytometry on cells collected non-enzymatically using a cell scraper, which did result in detection of CD90 expression. This suggests that the lack of CD90 expression using flow cytometry is possibly due to the trypsin-EDTA cleaving protein-related attachments, which, in turn, might cleave some epitope sites for binding the antibody [79]. It has been shown that in some cases, fluorescent FITC signal in flow cytometry decreases when cells are collected using trypsin-EDTA compared to other methods, such as accutase or cell scraper [80].

Moreover, we gave special consideration to the shipment conditions of MSCs to clinics within the country, and hence, we tested the viability of cAD-MSCs over 75 h under conditions mimicking the shipping of a package. Cellular viability of over 85% was maintained in saline for 48 h before viability decreased to 62.5% after 75 h. The viability results of the cAD-MSCs were not affected by temperatures exceeding 50 °C. The stability of the cAD-MSCs under these shipping conditions suggests the cells may survive shipping to clinics for therapeutic treatment. 

Finally, thinking proactively about the application of cAD-MSCs in tissue engineering projects, we demonstrated the expression of ECM proteins, including collagen I, collagen II, fibronectin, vinculin, and vimentin. Cells expressed and laid down the ECM within 24 h of attachment to the tissue culture substrate. F-actin staining proved the healthy cytoskeletal morphology of the cells. Data suggests that cAD-MSCs have the potential to express ECM and can be used in future tissue engineering strategies in conjunction with biomimetic scaffolds or biomaterials for the healing and repair of injured tissues.

## 5. Conclusions

Mesenchymal stem cells are derived from either adipose tissue or bone marrow and are expanded ex vivo in a tissue culture dish. This process is important for producing enough numbers of cells to be used in the clinic. Since cells are expanded in culture, it is important to characterize them to ensure that the cell culture process does not affect their potential as MSCs. We present general guidelines for MSC research and production. We believe that a novel MSC line should be characterized with respect to the criteria listed in Table 4, before they can be used safely and efficaciously in an allogenic manner in the clinic. 

## Figures and Tables

**Table 1:** List of antibodies used to prove the expression of MSC markers. Listed are the names, catalog numbers, companies, and references. Note that CD44 and CD45 were validated in this study.

Antibodies	Catalog Number	References
CD90	BioRad (Hercules, CA, USA) #MCA1036GA	[55]
MHCI	WSU Monoclonal Antibody Center (Pullman, WA, USA) #R-BOV2001	[55]
CD44	BioRad #MCA4703PE	[This study]
CD45	BioRad #MCA1042PB	[56]
MHCII	Invitrogen #11-5909-42	[55]

**Table 2:** List of antibodies used in immunofluorescence with the names, catalog numbers, companies and references.

Antibodies	Catalog Number	References
CD29	Novus (Littleton, CO, USA) #NB100-63255	[This Study]
CD44	BioRad #MCA4703PE	[This Study]
CD90	BioRad #MCA1036GA	[59]
Collagen I	abcam #ab34710 (Cambridge, UK)	[58]
Collagen II	abcam #ab34712	[58]
Fibronectin	R&D Systems #MAB19182 (Minneapolis, MN, USA)	[58]
Vimentin	BD Bioscience 550513 (New York, NY, USA)	[58]
Vinculin	abcam #ab129002	[58]

**Table 3:** Expression of specific MSC markers. Listed are the methods used to evaluate the expressions of cAD-MSC-specific markers. “+” shows positive expression, and “N/A” shows that marker expressions were not detected using that specific technique.

Cell Surface Markers	cAD-MSC Flow Cytometry (0.25% Trypsin/Cell Scraper)	Peripheral Blood (Heparin)	cAD-MSC Immunofluorescence
CD29	N/A	N/A	+
CD90	2.86%	77.61%	+
	55.30%		
CD44	67.95%	97.22%	+
	93.51%		
CD45	0.96%	99.31%	N/A
MHC I	0.04%	98.66%	N/A
MHC II	0.04%	20.642%	N/A

**Table 4:** Cryopreservation criteria and testing prior to use of cAD-MSCs clinically.

Specification	Expected	Method
Viability	≥90%	Trypan blue staining
Phenotype (CD29, CD44, CD45, CD90, MHCI, and MHCII)	≥95% (CD29, CD44, CD90) ≤1% (MHC I, MHC II, CD45)	Flow Cytometry/Immunofluorescence
Differentiation Potential	Osteogenesis, adipogenesis,	Induction culture

## Data Availability

The original contributions presented in the study are included in the article, further inquiries can be directed to the corresponding author.
